# External Validation of Models for Prediction of Lymph Node Metastasis in Urothelial Carcinoma of the Bladder

**DOI:** 10.1371/journal.pone.0120552

**Published:** 2015-10-01

**Authors:** Ja Hyeon Ku, Myong Kim, Seok-Soo Byun, Hyeon Jeong, Cheol Kwak, Hyeon Hoe Kim, Sang Eun Lee

**Affiliations:** 1 Department of Urology, Seoul National University Hospital, Seoul, Korea; 2 Department of Urology, Seoul National University Bundang Hospital, Seongnam, Korea; 3 Department of Urology, SMG-SNU Boramae Medical Center, Seoul, Korea; Centro Nacional de Investigaciones Oncológicas (CNIO), SPAIN

## Abstract

**Purpose:**

To externally validate models to predict LN metastsis; Karakiewicz nomogram, clinical nodal staging score (cNSS), and pathologic nodal staging score (pNSS) using a different cohort

**Materials and Methods:**

Clinicopathologic data from 500 patients who underwent radical cystectomy and pelvic lymphadenectomy were analyzed. The overall predictive values of models were compared with the criteria of overall performance, discrimination, calibration, and clinical usefulness.

**Results:**

Presence of pN+ stages was recorded in 117 patients (23.4%). Agreement between clinical and pathologic stage was noted in 174 (34.8%). Based on Nagelkerke’s peudo-R^2^ and brier score, pNSS demonstrated best overall performance. Area under the receiver operating characteristics curve, showed that pNSS had the best discriminatory ability. In all models, calibration was on average correct (calibration-in-the-large coefficient = zero). On decision curve analysis, pNSS performed better than other models across a wide range of threshold probabilities.

**Conclusions:**

When compared to pNSS, current precystectomy models such as the Karakiewicz nomogram and cNSS cannot predict the probability of LN metastases accurately. The findings suggest that the application of pNSS to Asian patients is feasible.

## Introduction

Radical cystectomy with lymph node (LN) dissection constitutes the standard treatment for muscle invasive and refractory nonmuscle invasive bladder cancer. As nodal disease is a powerful predictor of cancer-specific survival [1], knowledge of nodal status influences patient counseling and, more importantly, clinical decision making regarding follow-up scheduling and adjuvant chemotherapy [2,3].

Karakiewicz et al. [4] developed a multivariate nomogram with the intent of accurately predicting presence of LN metastases at cystectomy. Some investigators hypothesized that true nodal status could be accurately predicted based on the number of LNs examined and clinical or pathologic features; clinical nodal staging score (cNSS) [5] and pathologic nodal staging score (pNSS) [6] were developed to predict the probability that a patient with pathologically confirmed negative LNs is free of missed LN metastasis.

The aim of the present study was to externally validate the Karakiewicz nomogram, cNSS, and pNSS using a cohort from three centers from different countries.

## Materials and Methods

### Study group

Studies were undertaken with the approval of the Institutional Review Board (IRB) of Seoul National University Hospital (No. H-1212-057-450), Seoul National University Bundang Hospital (No. B-1310-222-114), and SMG-SNU Boramae Medical Center (No. 16-2013-127). All information of patients was anonymised and de-identified prior to analysis. The need for informed consent was waived by the IRB because of the retrospective design of this study. The medical records of patients who underwent radical cystectomy and pelvic lymphadenectomy at three medical centers in Korea were reviewed. For databases, detailed information of patient characteristics and pathologic details were collected. All identified data inconsistencies and integrity problems were resolved before analysis. We excluded patients <18-years-of-age, those presenting metastatic disease, and those with malignancies other than urothelial carcinoma. This study comprised 388 patients from Seoul National University Hospital, Seoul, Korea, between 1991 and 2011, 90 patients from Seoul National University Bundang Hospital, Seongnam, Korea between 2003 and 2011, and 22 patients from SMG-SNU Boramae Medical Center, Seoul, Korea between 2008 and 2011.

Clinical stage was assigned based on the pathologic evaluation of the transurethral resection (TUR) specimen, bimanual examination, and imaging results. Pathologic specimens were processed and evaluated according to standard pathologic procedures by staff surgical pathologists at each institution. Pathologic stage was assigned according to the 2002 American Joint Cancer Committee TNM classification [7]. Tumor grade was assessed according to the 1973 World Health Organization classification [8]. Lymphovascular invasion (LVI) was defined as the unequivocal presence of tumor cells in an endothelium lined space without underlying muscular walls. Positive surgical margins were defined as the microscopic presence of malignant cells at the resection margins.

### Predictive models

An online version of risk calculator developed by Karakiewicz et al. [4] is available as an on-line tool at http://labs.fccc.edu/nomograms/nomogram.php?id=40&audience=1&status=1. The Karakiewicz nomogram includes TUR stage (Ta-is-1/T2/T3), TUR grade (1-2/3), and pre-operative carcinoma *in situ* (absent/present). Each patient was tested with this on-line tool. After data were entered, the software calculated the probability of LN metastasis after radical cystectomy. cNSS is a look-up table requiring clinical T stage and number of LN retrieved [5]. pNSS is also a look-up table, which requires pathologic T stage, number of LNs retrieved, and the status of LVI [6].

### Statistical analyses

The overall predictive values of models were compared with several criteria. The overall performances of models (number of LNs removed, Karakiewicz nomogram, cNSS, and pNSS) were assessed separately by using R^2^ statistic (Nagelkerke’s pseudo-R^2^) [9] and Brier score (mean squared prediction error) [10]. Nagelkerke’s peudo-R^2^ can vary from 0 to 1, with a larger R^2^ indicating better predictive performance. Brier score was calculated for each patient and then averaged. A score of 0 indicates that the model can perfectly forecast patient-level outcomes, while the worst score achievable is 1.

Discrimination means the ability of the risk prediction models to distinguish those with event from those without event. Discriminative ability was determined by the area under the ROC curve. A score of 1 suggests that the model can perfectly discriminate between patients who will have LN metastases. A score of 0.5 indicates that the model has no discriminative ability. All area under the ROC curve estimates were internally validated using 500 bootstrap samples. Statistical differences in area under the ROC curves were evaluated by the nonparametric method [11].

Calibration means how closely the predicted probabilities reflect actual risk. We assessed general calibration by using a calibration plot. The relationship between the model-derived and actuarial outcome was graphically explored within calibration plots to explore model performance. The validation was done using 200 bootstrap resamples to decrease overfit bias. The calibration plot was characterized by an intercept, which indicates the extent that predictions are systemically too or too high, and calibration slope, which should be 1 [12,13]. A value of calibration slope may be interpreted as reflecting a need for shrinkage of regression coefficients in a prediction model [14].

Decision curve analysis (DCA) was used to explore the clinical value of each model [15]. DCA is a method for evaluating the clinical net benefit of prediction models; one sums the benefits (true positives) and subtracts the harms (false positives).

For all statistical analyses, two-sided p<0.05 was regarded as significant. Models, statistics, and Figs were prepared using SPSS software (SPSS, Chicago, IL) and R 2.13.2 (http://www.cran.r-project.org).

## Results

### Patient population

The demographic data for model development cohorts in comparison to external validation cohort is shown in Table 1. In model development cohorts, more than 40% of patients had locally advanced disease (pT3 or pT4) and about 25% exhibited LN-positive cancer (pN+). While no patients received neoadjuvant chemotherapy in cNSS and pNSS development cohort, neoadjuvant chemotherapy was administered in 4% of the nomogram development cohort and 9% of the external validation cohort.

**Table 1:** Clinical and pathologic characteristics of patients treated with radical cystectomy for urothelial carcinomas of the urinary bladder.

Variables	Nomogram development cohort	cNSS and pNSS development cohort	External validation cohort
No. of patients	726	4,335	500
Period	1984–2003	1980–2008	1991–2011
Age (years)			
Mean (median)	64.6 (66.0)	NA (67.0)	62.8 (64.0)
Range	33.8–89.2	23.0–93.0	25.0–85.6
Gender			
Male	593 (81.7%)	3,464 (80.0%)	455 (91.0%)
Female	133 (18.3%)	871 (20.0%)	45 (9.0%)
TUR T stage			
Tis	80 (11.0%)	NA	23 (4.6%)
Ta	16 (2.2%)	NA	37 (7.4%)
T1	173 (23.8%)	NA	149 (29.8%)
T2	375 (51.7%)	NA	277 (55.4%)
T3	45 (6.2%)	NA	0 (0.0%)
T4	37 (5.1%)	NA	14 (2.8%)
TUR grade			
1	7 (1.0%)	NA	4 (0.8%)
2	61 (8.4%)	NA	135 (27.0%)
3	658 (90.6%)	NA	361 (72.2%)
Concomitant CIS at TUR	294 (40.5%)	NA	53 (10.6%)
Clinical T stage*			
Tis	NA	316 (7.3%)	16 (3.2%)
Ta	NA	138 (3.2%)	18 (3.6%)
T1	NA	1,114 (25.7%)	102 (20.4%)
T2	NA	2,450 (56.5%)	249 (49.8%)
T3/4	NA	317 (7.3%)	115 (23.0%)
Pathologic T stage			
pT0/is/a	165 (19.7%)	774 (17.9%)	99 (19.8%)
pT1	91 (12.5%)	585 (13.5%)	73 (14.6%)
pT2	166 (22.9%)	1,042 (24.0%)	123 (24.6%)
pT3/4	304 (41.9%)	1,934 (44.6%)	205 (41.0%)
Pathologic N stage			
Negative	533 (76.2%)	3,216 (74.2%)	393 (76.6%)
Positive	173 (23.8%)	1,119 (25.8%)	117 (23.4%)
Presence of LVI at cystectomy	NA	1,475 (34.0%)	183 (36.6%)
Positive soft tissue surgical margin	NA	262 (6.1%)	35 (7.0%)
No. of examined LNs			
Mean (median)	NA	NA (18.0)	14.4 (13.0)
Range	NA	1–136	2–57
Neoadjuvant chemotherapy	38 (5.2%)	0 (0.0%)	45 (9.0%)
Neoadjuvant radiotherapy	NA	0 (0.0%)	0 (0.0%)

cNSS: clinical nodal staging score, pNSS: pathologic nodal staging score, NA: not available, TUR: transurethral resection, CIS: carcinoma in situ, LVI: lymphovascular invasion, LN: lymph node

*based on TUR T stage, bimanual examination and imaging study results.

Table 2 shows the cross-tabulation between clinical and pathologic stages. Overall, 205 patients (41.0%) had pT3-4 stages at cystectomy. Presence of pN+ stages was recorded in 117 patients (23.4%). Agreement between TUR and cystectomy stage was recorded in 135 (27.0%), while that between clinical and cystectomy stage was noted in 174 (34.8%). Of all patients, 258 (51.6%) and 175 (35.0%) had lower stage at TUR and clinical stage than at cystectomy, respectively. Conversely, stage reduction, which implies lower stage at cystectomy than at TUR and clinical stage, was noted in 107 (21.4%) and 151 (30.2%), respectively. Of 117 patients with LN metastases at cystectomy, T1 or lower disease at TUR was found in 34 (30.5%), T2 disease in 78 (28.2%), and T4 stages in5 (35.7%), whereas clinical T1 or lower disease was noted in 17 (22.0%), clinical T2 disease in 63 (25.3%), clinical T3 disease in 24 (32.0%), and clinical T4 disease in 13 (32.5%).

**Table 2:** Cross-tabulation between T stage at transurethral resection and clinical T stage and pathologic stage at cystectomy.

	Pathology at cystectomy							pN+	Total
	pT0	pTis	pTa	pT1	pT2	pT3	pT4		
TUR T stage									
Tis	5 (21.7%)	9 (39.1%)	0 (0.0%)	2 (8.7%)	4 (17.4%)	1 (4.3%)	2 (8.7%)	1 (4.3%)	23 (4.6%)
Ta	2 (5.4%)	3 (8.1%)	7 (18.9%)	11 (29.7%)	7 (18.9%)	4 (10.8%)	3 (8.1%)	2 (5.4%)	37 (7.4%)
T1	14 (9.4%)	13 (8.7%)	4 (2.7%)	38 (25.5%)	39 (26.2%)	27 (18.1%)	14 (9.4%)	31 (20.8%)	149 (29.8%)
T2	26 (9.4%)	11 (4.0%)	4 (1.4%)	19 (6.9%)	73 (26.4%)	124 (44.8%)	20 (7.2%)	78 (28.2%)	277 (55.4%)
T4	0 (0.0%)	1 (7.1%)	0 (0.0%)	3 (21.4%)	0 (0.0%)	2 (14.3%)	8 (57.1%)	5 (35.7%)	14 (2.8%)
Clinical T stage*									
Tis	5 (31.3%)	7 (43.8%)	0 (0.0%)	2 (12.5%)	0 (0.0%)	1 (6.3%)	1 (6.3%)	1 (6.3%)	16 (3.2%)
Ta	2 (11.1%)	3 (16.7%)	4 (22.2%)	7 (38.9%)	1 (5.6%)	0 (0.0%)	1 (5.6%)	0 (0.0%)	18 (3.6%)
T1	13 (12.7%)	8 (7.8%)	3 (2.9%)	29 (28.4%)	28 (27.5%)	13 (12.7%)	8 (7.8%)	16 (15.7%)	102 (20.4%)
T2	22 (8.8%)	15 (6.0%)	3 (1.2%)	23 (9.2%)	77 (30.9%)	95 (38.2%)	14 (5.6%)	63 (25.3%)	249 (49.8%)
T3	5 (6.7%)	3 (4.0%)	5 (6.7%)	7 (9.3%)	13 (17.3%)	38 (50.7%)	4 (5.3%)	24 (32.0%)	75 (15.0%)
T4	0 (0.0%)	1 (2.5%)	0 (0.0%)	5 (12.5%)	4 (10.0%)	11 (27.5%)	19 (47.5%)	13 (32.5%)	40 (8.0%)
pN+	4 (8.5%)	0 (0.0%)	0 (0.0%)	5 (6.8%)	22 (17.9%)	62 (39.2%)	25 (51.1%)	117 (23.4%)	
Total	47 (9.4%)	37 (7.4%)	15 (3.0%)	73 (14.6%)	123 (24.6%)	158 (31.6%)	47 (9.4%)		500 (100.0%)

TUR: transurethral resection

*based on TUR T stage, bimanual examination and imaging study results.

### Model performance

Data of model performances are presented in Table 3. pNSS demonstrated good predictive efficacy. Estimates of Nagelkerke’s peudo-R^2^ of pNSS were higher than those of others. The brier score of pNSS was 0.1482, which was lower than that of other models.

**Table 3:** Performance of models.

	No. of lymph nodes removed	Karakiewicz nomogram	Clinical nodal staging score	Pathologic nodal staging score
Overall				
Nagelkerke’s peudo-R^2^	0.7%	2.6%	2.1%	23.8%
Brier score	0.1782	0.1761	0.1768	0.1482
Discrimination				
Area under the curve (95% CI)*	0.514 (0.452–0.577)	0.588 (0.534–0.642)	0.589 (0.531–0.647)	0.776 (0.729–0.824)
Calibration				
Calibration-in-the-large	0	0	0	0
Calibration slope	1	1	1	1

CI: confidence interval

*p <0.001 for pathologic nodal staging score versus other models.

### Discrimination

Of models, pNSS had the highest bootstrap-corrected predictive accuracy (area under the ROC curve, 0.776; 95% confidence interval, 0.729–0.824). The bootstrap-corrected accuracies of other models were <60% (Table 3). The area under the ROC curve between pNSS and other models were statistically significant different (p <0.001).

### Calibration

**Fig 1:**
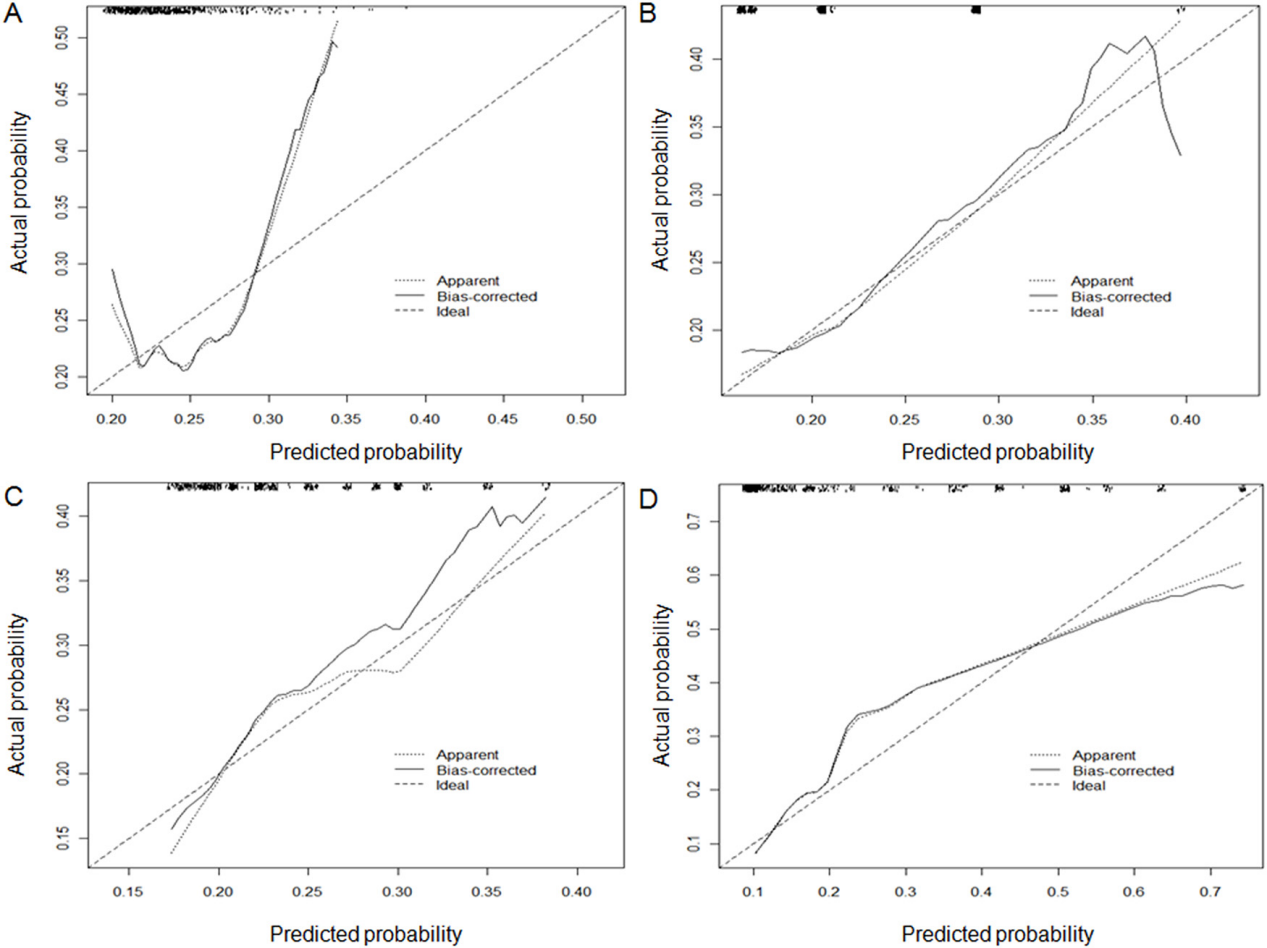
Calibration plots. (A) Number of lymph nodes removed. (B) Karakiewicz nomogram. (C) Clinical nodal staging score. (D) Pathologic nodal staging score.

In all models, calibration was on average correct (calibration-in-the-large coefficient = zero), and the effects of predictors were also on average correct in the new setting (calibration slope = 1) (Table 3). However, the calibration plots in Fig 1 demonstrated an underestimation of LN metastases. Even the calibration curve of pNSS did not perfectly match the line of identity (the line at a 45° angle) although the deviation was pictorially minimal.

### Clinical usefulness

**Fig 2:**
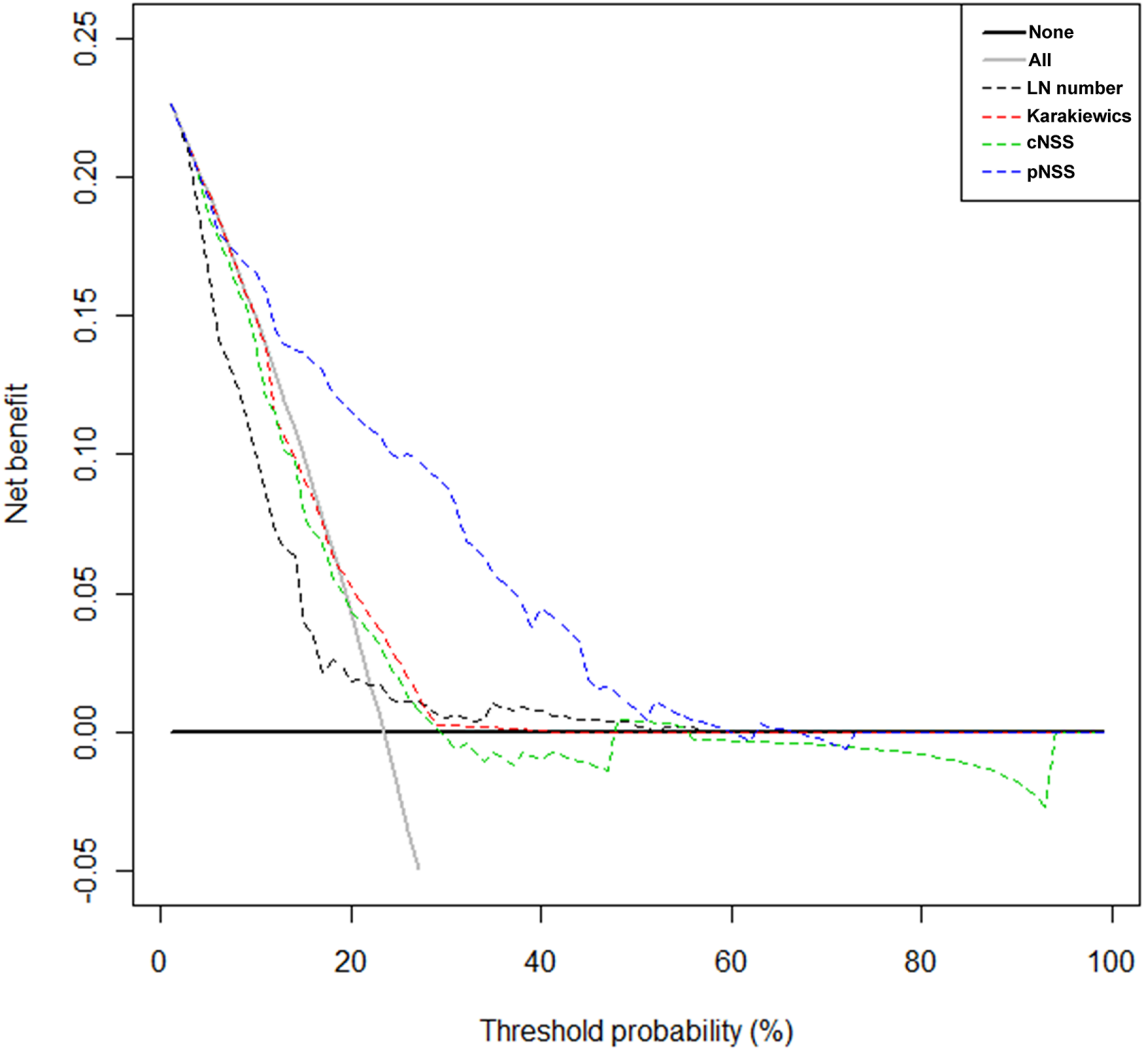
Decision curve analysis. Decision curve analysis. LN number = number of lymph nodes removed. Karkiewics = Karakiewicz nomogram. cNSS = clinical nodal staging score. pNSS = pathologic nodal staging score.

Fig 2 presents the results of the DCA. pNSS performed better than other models across a wide range of threshold probabilities.

## Discussion

Nodal status is a powerful predictor of bladder cancer recurrence and survival after radical cystectomy [16,17]. The rate of LN metastasis increases from a low of 5–10% in non-muscle invasive bladder tumors (≤pT1) to 15–20% in superficial muscle invasive tumors (pT2a), to 25–30% in deep muscle invasive tumors (pT2b), and to >40% in extravesical tumors (pT3-4) [4,16,18,19]. The probability of missing a positive LN may decrease with increasing number of LNs retrieved. Conversely, if a patient is LN-negative after only a few nodes have been examined, the likelihood of understanding is nontrivial. However, generally, the extent of lymphadenectomy is performed based on the surgeon’s intuitive experience integrating his beliefs and patient factors such as health status and tumor features [5]. Furthermore, although researchers have tried to identify the minimum necessary number of LNs needed to be removed at radical cystectomy, no minimum number of LNs can be determined [20].

In an effort to reduce staging errors, many experts have developed the models predicting true nodal status (no false-negative LN status) in bladder cancer [4–6]. The Karakiewicz nomogram represents the first attempt at defining objective, systematic, standardized, multivariate models capable of providing individual pN stage predictions [4]. cNSS is a simple probabilistic model to predict the number of LNs needed to be removed as a function of clinical stage [5]. pNSS is a simple probabilistic model that calculates the probability of freedom from missed LN metastasis as a function of pathologic tumor stage and LVI [6].

To introduce these predictive tools into the daily patient care in different continents, they must be externally validated in a variety of data sets, since external validation represents the gold standard for assessing the ability of staging tools to discriminate between those with and without the end point of interest. Karakiewicz nomograms failed to retain favorable discrimination ability in a European series because LN involvement was underestimated in an external dataset [21]. May et al. [21] applied the Karakiewicz nomogram in 2,477 German patients. The authors found that the Karakiewicz nomogram for LN metastasis underestimated the incidence of LN metastasis (54.5% accuracy). Even in the original paper, the maximum accuracy of the Karakiewicz nomogram for pN+ predictions was 63.3%, which implies that 36.7% of patients would still be misclassified [4].

Gierth et al. [22] assessed 2,483 patients in eight German tertiary centers to validate cNSS and pNSS. The validation of cNSS and pNSS was performed using a beta-binomial model in the same manner as described previously [5,6]. The authors found that the external validation of both scores yielded LN number closely reflecting other results [5,6]. Our previous study results also support the view that cNSS is superior to the number of LNs removed in terms of its prognostic value in patients without LN metastasis [23]. When probability of missing positive LN of <10% (cNSS 90%) was set, the accuracy of multivariate Cox regression model was 0.761 at 5 years. However, it remains unknown whether a correlation between a model and survival reflects improved LN staging accuracy.

The aim of the present study was to externally validate predictive models for LN metastasis in a different cohort of patients who had undergone radical cystectomy. The applicability of models derived from cohorts in North America and Europe may be affected when transferred to Asian cohorts. Only pNSS performed adequately within this external cohort of patients, and this finding was consistent using different statistical means (i.e., overall performance, discrimination, calibration, and clinical usefulness). In our study, discrepancy between clinical and pathologic stage was common in patients who undergo radical cystectomy; our findings indicate an agreement between the clinical and pathologic stage in 34.8% of patients. This discrepancy is also shown in other previous study [24] and may be the result of the retrospective collection of patient data. Studer and Sylvester [25] criticized the number of LNs defined sufficient by cNSS, since an important confounding and unquantifiable factor is the clinical staging error.

The limitations of the present study are inherent to any retrospective series. Lymphadenectomy templates were not standardized. Although LN count is probably closely correlates with extent of dissection, it is not the ideal proxy for the extent of lymphadenectomy. In addition, the number of LNs may be different in any given individuals and dependent on pathologic evaluation. Moreover, in the present study, central pathology review was not performed. Therefore, there may be potential risks for inter-observer differences in final pathologic results. Conversely, our data reflects a real-world multicenter experience and pathologic examination was performed by genitourinary pathologists in major academic centers [5,6]. Performing an adequate lymphadenectomy with adherence to meticulous dissection of LNs may be more important than achieving a minimal LN count.

Since thee models have been designed to test different concept, it may not be suitable to compare their performance. In addition, the Karakiewicz nomogram included patients who had received neoadjuvant chemotherapy, but both cNSS and pNSS did not. Since patients who had received neoadjuvant chemotherapy were included in the present analysis, neoadjuvant chemotherapy might influence the results. However, we could observe similar findings in the cohort without neoadjuvant chemotherapy (data not shown). Finally, pNSS was provided as a look-up table. Although a simple model like look-up table is easier to understand, it might have an inferior predictive accuracy compared with nomograms.

## Conclusions

As there is a significant discrepancy between clinical and pathologic stage, current precystectomy models based on clinical stage might not be applicable for prediction of LN metastasis. Our findings suggest that current precystectomy models for prediction of LN metastasis should be improved further. Conversely, our findings encourage the use of pNSS for prediction of LN metastasis of Asian patients.
